# Calcium Handling in Inherited Cardiac Diseases: A Focus on Catecholaminergic Polymorphic Ventricular Tachycardia and Hypertrophic Cardiomyopathy

**DOI:** 10.3390/ijms24043365

**Published:** 2023-02-08

**Authors:** Stéphane Zaffran, Lilia Kraoua, Hager Jaouadi

**Affiliations:** 1 Marseille Medical Genetics, INSERM, Aix Marseille University, U1251 Marseille, France; 2 Department of Congenital and Hereditary Diseases, Charles Nicolle Hospital, Tunis 1006, Tunisia

**Keywords:** catecholaminergic polymorphic ventricular tachycardia, excitation–contraction coupling, hypertrophic cardiomyopathy, calcium mishandling

## Abstract

Calcium (Ca^2+^) is the major mediator of cardiac contractile function. It plays a key role in regulating excitation–contraction coupling and modulating the systolic and diastolic phases. Defective handling of intracellular Ca^2+^ can cause different types of cardiac dysfunction. Thus, the remodeling of Ca^2+^ handling has been proposed to be a part of the pathological mechanism leading to electrical and structural heart diseases. Indeed, to ensure appropriate electrical cardiac conduction and contraction, Ca^2+^ levels are regulated by several Ca^2+^-related proteins. This review focuses on the genetic etiology of cardiac diseases related to calcium mishandling. We will approach the subject by focalizing on two clinical entities: catecholaminergic polymorphic ventricular tachycardia (CPVT) as a cardiac channelopathy and hypertrophic cardiomyopathy (HCM) as a primary cardiomyopathy. Further, this review will illustrate the fact that despite the genetic and allelic heterogeneity of cardiac defects, calcium-handling perturbations are the common pathophysiological mechanism. The newly identified calcium-related genes and the genetic overlap between the associated heart diseases are also discussed in this review.

## 1. Introduction

In the heart, calcium (Ca^2+^) is fundamental for the generation of the contractile force that gives rise to the heartbeat. Indeed, Ca^2+^ plays a key role in excitation–contraction coupling (ECC) and electrophysiological signaling in the heart [1]. Of note, the sarcoplasmic reticulum (SR) acts as an intracellular Ca^2+^ storage in the cardiomyocytes. The regulation of the release of Ca^2+^ ions from SR Ca^2+^ stores is mediated by the ECC, which then propagates and binds to the myofilaments to initiate the systole phase (contraction). The diastole phase (relaxation) is then ensured by the reuptake of Ca^2+^ into the SR Ca^2+^ store via sarcoendoplasmic reticulum Ca^2+^ ATPase (SERCA2a) or extruded from the cell via the Na^+^/Ca^2+^ exchanger [1,2,3]. This process is known as Ca^2+^-induced Ca^2+^ release (CICR) and is mediated by the ryanodine receptors (Ca^2+^ activation of Ca^2+^ release channels) [4]. Intracellular Ca^2+^ homeostasis in cardiomyocytes is regulated by the phosphorylation and dephosphorylation of several key Ca^2+^-handling proteins.

Given that Ca^2+^ is the cornerstone of cardiac electrophysiology and contraction, calcium mishandling has been associated with contractile dysfunction, arrhythmia, and cellular hypertrophy [5]. 

In this review, we focus on the genetic etiology of cardiac diseases related to calcium mishandling. We will approach the subject by focalizing on two clinical entities: catecholaminergic polymorphic ventricular tachycardia (CPVT) as a cardiac channelopathy and hypertrophic cardiomyopathy (HCM) as a primary cardiomyopathy. HCM is considered the most common primary cardiomyopathy, usually due to heterozygous mutations in sarcomeric genes. HCM may lead to severe complications such as heart failure and sudden cardiac death (SCD). On the other hand, catecholaminergic polymorphic ventricular tachycardia (CPVT) is a rare genetic arrhythmia that is mainly defined by bidirectional or polymorphic ventricular tachycardia occurring during physical activity or emotional stress. CPVT patients are predisposed to life-threatening arrhythmias. CPVT is caused by mutations in Ca^2+^-regulating genes. This review will define both diseases and describe their genetic etiology by reporting the different Ca^2+^-related genes.

## 2. Methods

We conducted a literature search on PubMed for cardiac disorders related to calcium handling with a special focus on catecholaminergic polymorphic ventricular tachycardia (CPVT) and hypertrophic cardiomyopathy (HCM). Different keyword combinations were applied: “catecholaminergic polymorphic ventricular tachycardia” AND “calcium” OR “calcium handling” OR “calcium mishandling” OR “calcium homeostasis”, “catecholaminergic polymorphic ventricular tachycardia” AND “RYR2, CASQ2, TECRL, TRDN, CALM1, CALM2, CALM3, PKP2 OR ANK2”,“hypertrophic cardiomyopathy” AND “calcium” OR “calcium handling” OR “calcium mishandling” OR “calcium homeostasis”, “hypertrophic cardiomyopathy” AND “calcium” OR “calcium handling” OR “calcium mishandling” OR “calcium homeostasis”, “hypertrophic cardiomyopathy” AND “RYR2, ALPK3, TNNC1, JPH2 OR PLN”. The relevance of the articles was determined by screening titles and abstracts. About sixty articles were selected for each heart disease. 

Gene data, namely the gene symbol, location, exon count, protein function, and associated OMIM diseases, were collected from several databases: NCBI-gene (https://www.ncbi.nlm.nih.gov; accessed 15 September 2022), Uniprot (https://www.uniprot.org; accessed 15 September 2022), GeneCards (https://www.genecards.org/; accessed 15 September 2022), GeneMANIA (https://genemania.org; accessed 2 November 2022), and OMIM (https://www.omim.org; accessed 2 November 2022).

All variants were described following the recommendations of the Human Genome Variation Society (http://varnomen.hgvs.org/recommendations; accessed 22 January 2023).

## 3. Results and Discussion

### 3.1. Catecholaminergic Polymorphic Ventricular Tachycardia

CPVT is a genetic stress-induced cardiac channelopathy characterized by adrenergically mediated polymorphic ventricular tachyarrhythmias that may lead to SCD, particularly in pediatric and young adult cases [6,7,8]. As the name implies, CPVT is triggered by increased catecholamines during exercise or emotional stress in healthy individuals with structurally normal hearts and a regular resting ECG [6].

The prevalence of CPVT is about 1:10,000 in the general population [8,9]. The average age of onset of clinical symptoms is between 7 and 9 years [7,10]. 

Clinically, CPVT is diagnosed in the absence of structural cardiac anomalies, normal ECG, and unexplained catecholamine-induced or stress-induced bidirectional VT, polymorphic premature ventricular beats, or VT in persons younger than 40 years [11].

Usually, a definite CPVT diagnosis is made after an average delay of 2 years from the first arrhythmogenic event due to a primary suspicion of vasovagal discomfort or a neurological etiology [12]. The early detection of CPVT is crucial to prevent SCD. Hence, molecular genetic screening is necessary to confirm an uncertain clinical diagnosis of CPVT and identify asymptomatic family members.

The life-threatening arrhythmias in CPVT are mainly caused by unregulated Ca^2+^ release from the SR [13]. Indeed, the electrocardiographic pattern of the ventricular tachycardia observed in CPVT patients is very similar to the arrhythmias linked to intracellular Ca^2+^ overload and the delayed afterdepolarizations (DADs) detected in digitalis toxicity [14,15]. Thus, DADs and triggered physical activity have been proposed as the underlying arrhythmogenic mechanism in CPVT [14,15]. Consistently, mutations in calcium-handling proteins implicated in the release of Ca^2+^ from the SR are associated with the CPVT phenotype, namely the RYR2 and CASQ2 proteins [3,16]. Of note, RYR2 and CASQ2 are parts of the multimolecular Ca^2+^ release channel complex located in the SR. Although RYR2 serves as a Ca^2+^ release channel, the SR Ca^2+^-binding protein, CASQ2, plays a dual role by regulating RYR2 function and serving as a buffer for SR Ca^2+^ [13].

To date, a handful of Ca^2+^-handling genes have been linked to CPVT including mainly the *RYR2* gene and, less frequently, the *CASQ2*, *TRDN*, *CALM1*, *CALM2*, *CALM3*, *TECRL*, *PKP2*, and *ANK2* genes [3,11]. Indeed, the incidence of *RYR2* mutations in CPVT patients ranges from 35 to 79%, whereas mutations in the *CASQ2* gene account for 3–5% of CPVT cases [11]. The CPVT-associated genes with their main functions are summarized in Table 1.

#### 3.1.1. Major CPVT Genes

##### Ryanodine Receptor 2

The ryanodine receptor 2 (*RYR2*) gene encodes the cardiac ryanodine receptor in the SR. The encoded RYR2 protein is the major Ca^2+^ channel protein in the membrane of the SR, which acts as intracellular Ca^2+^ storage in cardiomyocytes. Ca^2+^ can be released from the SR to the cytosol by the RYR2 channels [17]. Ca^2+^ release in cardiomyocytes is triggered by increased Ca^2+^ levels due to the activation of CACNA1C channels. A dysfunctional RYR2 channel leads to diastolic Ca^2+^ leak from the SR and contributes to the development of DADs [15].

During normal cardiac contraction, RYR2 is activated by cytosolic Ca^2+^, whereas under the conditions of storage overload, RYR2 opening is regulated by SR Ca^2+^ [18]. In addition to RYR2 activation, SR luminal Ca^2+^ also determines the RYR2 channel closing [18].

During exercise, RYR2 phosphorylation by protein kinase A partially dissociates FK-binding protein 12.6 (FKBP12.6) from the RYR2 channel, leading to an increase in intracellular Ca^2+^ release and cardiac contractility [19]. Functional studies using Fkbp12.6 −/− mice showed exercise-induced cardiac ventricular arrhythmias resulting in SCD. Indeed, *RYR2* mutations linked to exercise-induced arrhythmias in patients with CPVT reduced the affinity of FKBP12.6 for RYR2 channels and increased single-channel activity during exercise. These findings suggested that ‘leaky’ RYR2 channels can trigger malignant arrhythmias, likely causing CPVT [19]. 

Gain-of-function mutations in *RYR2* are found in approximately 79% to 95 % of CPVT1 cases with an autosomal dominant pattern of inheritance [11,20]. Loss-of-function *RYR2* mutations are less frequent and linked to other ventricular arrhythmia syndromes [7].

CPVT-linked *RYR2* mutations increase the likelihood of spontaneous RYR2 openings and Ca^2+^ leak from the SR during diastole, triggering malignant arrhythmias [20]. To date, over 150 *RYR2* mutations have been associated with CPVT [16]. The majority of *RYR2* mutations are located in four well-conserved domains including the pore, pseudo-voltage sensor, and central domains. Indeed, these domains are implicated in channel activation and gating. A potential link between mutation localization and phenotype severity has been emphasized [18,21]. Therefore, mutations located in the C-terminus of the RYR2 protein have been correlated with sudden death during sleep [22].

##### Calsequestrin 2

The calsequestrin 2 (*CASQ2*) gene encodes the calsequestrin protein localized in the SR of cardiac and slow skeletal muscle cells. CASQ2 is Ca^2+^-binding protein that stores Ca^2+^ for muscle function. Ca^2+^ ions are bound by clusters of acidic residues at the protein surface [23]. CASQ2 plays a pivotal role in triggering muscle contraction by regulating the release of lumenal Ca^2+^ through the RYR2 channel. Thus, CASQ2 significantly contributes to the cardiac ECC and regulates the heartbeat rate [23].

Mutations in *CASQ2* cause approximately 2 to 5% of CPVT with an autosomal recessive pattern of inheritance. Rarely is an autosomal dominant model associated with *CASQ2* mutations [8]. *CASQ2* c.539A>G; p.(Lys180Arg) was the first reported mutation associated with an autosomal-dominant inheritance of CPVT [24].

Mutations in *CASQ2* result in a lack of control of the RYR2 channel and, consequently, a constant release of Ca^2+^ into the cytoplasm, leading to arrhythmias [13]. Indeed, to regulate Ca^2+^ release, calsequestrin is anchored to RYR2 by triadin and junction proteins. It has been proposed that the interaction between CASQ2 and RYR2 may contribute to the refractory period of Ca^2+^ release occurring after each physiological CICR but the mechanism is not fully understood [13,20]. The most plausible CPVT mechanism linked to nonsense *CASQ2* mutations is impaired Ca^2+^ buffering. However, missense mutations, such as the *CASQ2* c.98G>A; p.(Arg33Gln), have been linked to Ca^2+^ buffering decrease and an alteration of CASQ2/RYR2 interaction [20].

*Casq2*-null mice exhibited normal Ca^2+^ release from the SR and contractile function under basal conditions. However, mutant mice had an increase in SR volume and an absence of Casq2-binding proteins such as triadin-1 and junction [25]. Exposure to catecholamines in Casq2-null myocytes induced increased diastolic SR Ca2+ leak, which resulted in premature spontaneous SR Ca^2+^ release and triggered arrhythmias [25].

##### Trans-2,3-Enoyl-CoA Reductase-like

The trans-2,3-enoyl-CoA reductase-like (*TECRL*) gene encodes the trans-2,3-enoyl-CoA reductase protein, which is an endoplasmic reticulum protein mainly expressed in the heart and skeletal muscle [26]. The TECRL protein consists of 363-amino acid with an N-terminal ubiquitin-like domain, 3 transmembrane regions, and a C-terminal 3-oxo-5-alpha steroid 4-dehydrogenase domain and plays a crucial role in intracellular Ca^2+^ homeostasis [27]. Indeed, the concentration of the expressed TECRL protein is critical for Ca^2+^ regulation of major cardiac proteins such as RYR2, CASQ2, and CALM [26,27].

Bhuiyan et al. in 2007 described the first CPVT phenotype associated with the *TECRL* gene according to an autosomal recessive pattern of inheritance, mapped to chromosome locus 7p22–p14 [28]. The members of this family were diagnosed with an early-onset and highly malignant form of CPVT with a history of SCD during physical activity [27,28]. Using exome sequencing, Devalla et al. (2016) identified a homozygous loss-of-function mutation in the *TECRL* gene in all the affected members of this same family [27].

To assess the functional consequence of the *TECRL* c.331+1G>A mutation, human induced pluripotent stem cells (hiPSCs) from a 5-year-old homozygous patient (TECRLHom-hiPSCs), his heterozygous father (TECRLHet-hiPSCs), and a non-carrier family member (CTRL-hiPSCs) were generated [27]. hiPSCs were differentiated into cardiomyocytes (CMs) and analyzed in vitro. Using this in vitro model, the authors showed that the c.331+1G>A mutation in *TECRL* leads to the skipping of exon 3. Moreover, the TECRLHomhiPSC-CMs closely replicated the disease phenotype and the mutant cells showed an increase in triggered electrical activity upon catecholaminergic stimulation [27]. 

Analysis of intracellular calcium dynamics of the TECRLHom-hiPSCs revealed altered Ca^2+^ properties, including a high diastolic Ca^2+^, smaller amplitude and slower decay of cytosolic Ca^2+^ transients, and a prolonged action potential duration [26,27]. This study reported two additional families with overlapping clinical features of long QT syndrome (LQTS) and CPVT carrying *TECRL* mutations [27]. Interestingly, both heterozygous and homozygous *TECRL* c.587G>A; p.(Arg196Gln) mutations can cause a decrease in Ca^2+^ stores in the SR and an increase in diastolic cytoplasmic Ca^2+^ concentration in cardiomyocytes, leading to a CVPT phenotype [27]. 

The *TECRL* c.331+1G>A mutation was subsequently reported by Jaouadi et al. (2020) in a consanguineous family with three deceased children, each at 8 years old [29]. The three SCD events occurred during normal daily activities (playing, slow walking, and at school). Exome sequencing of the family revealed the presence of the homozygous *TECRL* c.331+1G>A mutation in the last deceased child. Both parents were found to be heterozygous for the variant. The father was asymptomatic with a structurally normal heart and no history of cardiac arrhythmias, whereas the mother had a history of syncopes and a clinical suspicion of Brugada syndrome [29]. Intriguingly, no CPVT or LQTS features were noted in this family [29].

Although the first reported patients with *TECRL* mutations displayed strict CPVT features or CPVT-specific features combined with a long QT interval but not an isolated LQTS [27,30], the newly identified cases carrying *TECRL* mutations displayed divergent cardiac phenotypes within a single genetic locus [30].

Moscu-Gregor et al. (2020) have identified four additional mutations in the *TECRL* gene in CPVT patients with severe and early-onset clinical presentation at the homozygous and compound heterozygous state (c.415C>T; p.(Gln139*), c.893T>C; p.(Val298Ala), c.926C>A; p.(Ser309*), and c.869C>A; p.(Pro290His)). The authors concluded that variants in *TECRL* may be causative of up to 5% of CPVT patients [31].

Overall, patients with *TECRL* mutations presented a highly lethal form of arrhythmias, with a median age of symptom onset at 8 years of age [30,32]. 

##### Triadin

Triadin (TRDN) is one of the major transmembrane proteins located in the junctional SR playing a role in ECC regulation and Ca^2+^ influx in the calcium release complex [33]. Mutations in the *TRDN* gene lead to a significant decrease in protein expression causing Ca^2+^ overload in the SR, which may explain the development of CVPT [34,35]. 

From a cohort of 97 CPVT patients with no mutations in the *RYR2* and *CASQ2* genes, Roux-Buisson et al. (2012) identified three mutations in the *TRDN* gene, which cosegregated with the disease according to an autosomal recessive pattern in two families: a c.del53_56ACAG; p.(Asp18Alafs*13) homozygous deletion in the first family and compound heterozygous mutations c.176C>G; p.(Thr59Arg) and c.613C>T; p.(Gln205*) in the second family [35]. Thereafter, Rooryck et al. (2015) identified compound heterozygous pathogenic mutations (c.613C>T; p.(Gln205*) and c.22 + 29 A>G) in two sisters with CPVT [36]. Overall, the *TRDN* gene was associated with less than 1% of CPVT5 cases [35].

##### Calmodulin

Calmodulin proteins (CALM) are members of the EF-hand calcium-binding protein family and play an essential role in Ca^2+^ sensing and signal transducing. Three distinct calmodulin genes (*CALM1*, *CALM2*, and *CALM3*) are distributed within the human genome that encode the identical protein but differ at the nucleotide level. The three calmodulin genes share about an 80% identity within their coding regions. Calcium-induced activation of calmodulin modulates the function of cardiac ion channels including *CACNA1C*, *SCN5A*, and *RYR2* [37,38].

Mutations in *CALM1*, *CALM2*, and *CALM3* genes have been associated with CPVT.

Nyegaard et al. (2012) identified a heterozygous *CALM1* c.161A>T; p.(Asn53Ile) mutation that segregated with the disease in 10 affected family members [38]. A de novo missense mutation in *CALM1*, c.293A>G; p.(Asn97Ser), was subsequently identified in a 23-year-old woman with a history of resuscitated cardiac arrest at 4 years of age due to ventricular fibrillation while running. Both substitutions showed compromised calcium binding [38]. 

Makita et al. (2014) identified two heterozygous missense *CALM2* mutations in two patients with overlapping features of LQTS and CPVT [39]. The c.396T>G; p.(Asp132Glu) mutation was identified in a 29-year-old woman who was initially diagnosed with neonatal LQTS and later with exercise-induced polymorphic ventricular ectopy. The second *CALM2* variant, c.407A>C; p.(Gln136Pro), was identified in an 8-year-old girl with a presumptive diagnosis of LQTS and CPVT who died suddenly during exercise despite treatment with β-blockers [39]. The two mutations were de novo. The encoded mutant calmodulin proteins impaired C-domain Ca^2+^-binding affinity, likely causing Ca^2+^ signaling dysfunction [39]. 

Gomez-Hurtado et al. (2016) identified a heterozygous *CALM3* mutation, c.308C>T; p.(Ala103Val), in a 31-year-old woman among a cohort of 12 CPVT patients with no mutations in the other known CPVT genes. The *CALM3* mutation was shown to activate RYR2 Ca^2+^ release channels, generating Ca^2+^ waves and depleting the SR Ca^2+^ store [40,41]. Moreover, it has been shown that CPVT calmodulin mutants tend to bind to RYR2 with higher affinity than wild-type, which can explain their autosomal-dominant mode of action [40,41].

Thus, both de novo and inherited mutations have been reported and patients harboring *CALM* mutations may present overlapping features of LQTS and CPVT.

#### 3.1.2. Minor CPVT Genes

##### Plakophilin-2

The plakophilin-2 (*PKP2*) gene encodes the desmosomal plakophilin-2 protein. Mutations in the *PKP2* gene were associated primarily with arrhythmogenic right ventricular cardiomyopathy (ARVC) [42]. Subsequently, Cerrone et al. (2017) have used conditional mouse deletion of *Pkp2* in cardiomyocytes to demonstrate that the lack of Pkp2 reduces expression levels of Ryr2, Ank2, Cacna1c, Trdn, and Casq2 proteins, leading to disruption of intracellular Ca^2+^ homeostasis and isoproterenol-induced arrhythmias, even in the absence of overt structural heart disease [43]. Tester et al. (2019) screened the *PKP2* gene in genotype-negative patients with CPVT. *PKP2* mutations were found in 27.7% of CPVT cases and 5.3% of exercise-related sudden unexplained death in the young cases. Cardiac imaging or autopsy demonstrated a structurally normal heart in all patients [44]. 

##### Ankyrin-2

The ankyrin 2 (*ANK2*) gene encodes an ankyrin-B protein that is located mainly in the transverse-tubule SR sites of the cardiomyocytes [45]. This protein is a crucial part of the Na^+^/Ca^2+^ exchanger, Na^+^/K^+^ ATPase, and inositol trisphosphate (InsP3) receptor. Thus, ankyrins have key roles in membrane trafficking and regulation of different ion channels in the heart [46]. A loss-of-function mutation of ankyrin-B was initially associated with LQTS 4 [45]. Subsequently, Mohler et al., (2004 and 2007), identified nine *ANK2* loss-of-function mutations in patients with variable expressivity of cardiac dysfunction including bradycardia, sinus arrhythmia, idiopathic ventricular fibrillation, and CPVT [47,48]. The CPVT patients carried the *ANK2* c.4864C>A; p.(Leu1622Ile), c.5437G>A; p.(Glu1813Lys), and c.T4547A; p.(Val1516Asp) mutations [47,48]. The *ANK2* mutations were shown to abolish the ability of ankyrin-B to restore defective Ca^2+^ dynamics. The authors also noted abnormal localization and expression of the Na^+^/Ca^2+^ exchanger, Na^+^/K^+^ ATPase, and InsP3 [47]. 

**Table 1:** Calcium-related genes linked to catecholaminergic polymorphic ventricular tachycardia.

HGNC_Gene Symbol	Full Name	Location	Exon Count	Protein Function	OMIM Disease	OMIM IDs	Inheritance	References
*RYR2*	Ryanodine receptor 2	1q43	105	Calcium release channels from the sarcoplasmic reticulum into the cytoplasm by ER and SR. Activates and modulates small-conductance Ca^2+^-activated K+ channels in cardiac myocytes. Regulates cardiac muscle contraction by calcium ion signaling.	ARVD2	600996	AD	[18,49,50]
					VA	115000	AD	
					CPVT1	604772	AD	
*CASQ2*	Calsequestrin 2	1p13.1	11	Major Ca^2+^-binding protein in the SR. Key SR Ca^2+^ storage protein essential for SR Ca2+ release in the heart. Structural organization of the SR with *TRDN*. Facilitates high rates of Ca^2+^ release through RYR2 during systole. Plays a critical role in mobilizing Ca^2+^ release from ER/SR lumens. Role in ECC in the heart and regulation of heart rate beats. Regulates cardiac muscle conduction and contraction by calcium ion signaling. Regulates ryanodine-sensitive calcium-release channel activity.	CPVT2	611938	AR/ AD	[51,52]
*TECRL*	Trans-2,3-Enoyl-CoA Reductase Like	4q13.1	12	ER protein. Role in intracellular Ca^2+^ homeostasis. Regulates heart contraction.	CPVT3	614021	AR	[26,27,30,31]
*TRDN*	Triadin	6q22.31	41	Contributes to the regulation of lumenal Ca^2+^ release via the SR calcium release channels RYR1 and RYR2, a key step in triggering skeletal and heart muscle contraction. Regulates the release of sequestered calcium ions into the cytosol by SR. Cell–cell signaling involved in cardiac conduction. Anchors calsequestrin to the junctional SR, allowing its functional coupling with the ryanodine receptor. Indirect role of triadin in regulating myoplasmic Ca^2+^ homeostasis and organizing the molecular complex of the triad but not in regulating skeletal-type excitation–contraction coupling.	CPVT5	615441	AR	[35,53]
*CALM1*	Calmodulin 1	14q32.11	6	Intracellular Ca^2+^ transducer involved in numerous activities in a broad Ca^2+^ signaling network. Regulates RYR1 and RYR2 by binding to a single, highly conserved calmodulin binding site. Regulates tail-anchored insertion into the ER membrane in a Ca^2+^-dependent manner.	LQTS14	616247	AD	[54,55]
					CPVT4	614916	AD	
*CALM2*	Calmodulin 2	2p21	6	Phosphorylase kinase, delta, calcium-modulated protein. Mediates the control of a large number of enzymes and other proteins by Ca^2+^. Plays a crucial role in the processes of Ca2+-induced neuronal cell death.	LQTS 15	616249	AD	[37,56]
*CALM3*	Calmodulin 3	19q13.32	6	Calcium-modulated protein. Mediates the control of a large number of enzymes by Ca^2+^, protein kinases, and phosphatases.	CPVT6	618782	AD	[37,40,57]
					LQTS16			
*PKP2*	Plakophilin-2	12p11.21	13	Regulates the signaling activity of beta-catenin. Maintains the transcription of genes that control intracellular calcium cycling including *RYR2*, *ANK2*, *TRDN*, and *CACNA1C*. Regulates cardiac muscle cell contraction and cell action potential. Regulates actin filament-based movement/cardiac muscle tissue development/cell–cell junction organization.	ARVD 9	609040	AD	[42]
*ANK2*	Ankyrin 2	4q25-q26	46	Required for coordinated assembly of Na/Ca exchanger, Na/K ATPase, and inositol trisphosphate INSP3 receptor at transverse-tubule/sarcoplasmic reticulum sites in cardiomyocytes. Regulates cardiac muscle contraction by calcium ion signaling. Role in normal cardiac electric activity and cardiac automaticity. Regulates *KCNJ5* channel gating.	LQTS4	600919	AD	[58,59,60]

ARVD: arrhythmogenic right ventricular dysplasia; AD: autosomal dominant; AR: autosomal recessive; CPVT: catecholaminergic polymorphic ventricular tachycardia; DCM: dilated cardiomyopathy; ECC: excitation–contraction coupling ER: endoplasmic reticulum; SR: sarcoplasmic reticulum; HCM: hypertrophic cardiomyopathy; LQTS: long QT syndrome.

### 3.2. Hypertrophic Cardiomyopathy

HCM is a primary cardiac disorder characterized by an increased left ventricular wall thickness in the absence of other loading conditions [61]. HCM is the most common inherited heart disease, with a prevalence of 1/200 to 1/500, and is mostly inherited in an autosomal dominant manner [62,63]. Patients with HCM have a higher risk of developing clinical complications such as progressive heart failure, arrhythmia, and SCD [63]. Molecular genetic studies have demonstrated that HCM is mainly caused by mutations in sarcomeric genes encoding contractile myofilament proteins [62,64,65,66]. The frequency of mutations within sarcomeric genes varies from 25% to 65% of patients [67]. 

Increased calcium buffering has been proposed as the causal mechanism leading to the alteration of intracellular Ca^2+^ cycling and triggering Ca^2+^-dependent hypertrophy [68]. In vivo experiments using LV guinea pig cardiomyocytes expressing mutations in the *TNNT2*, *TNNI1*, and *TPM1* genes demonstrated increased diastolic Ca^2+^ and Ca^2+^ reuptake [68]. Moreover, mutations in the *MYBPC3* gene may increase myofilament Ca^2+^ sensitivity and promote cardiac hypertrophy due to the inability to release Ca^2+^ and relax from contraction [69].

Nevertheless, there is a lack of strong evidence to show whether these alterations are a causal factor of HCM or consequential clinical manifestations. With the advent of patient-specific iPSC models, Lan et al. (2013) have provided evidence that the elevation of intracellular Ca^2+^ is the initial factor in HCM development [5]. Indeed, time-based gene expression data analysis of single iPSC-CMs carrying the *MYH7*: Arg663His mutation revealed that the downstream effectors of cardiac hypertrophy (e.g., *GATA4* and *MEF2*) were expressed in a Ca^2+^-dependent manner [5]. Furthermore, the authors concluded that elevated cardiomyocyte Ca^2+^ loading seems to contribute to both cardiac hypertrophy and arrhythmogenesis [5].

Given the aforementioned observations and key role of Ca^2+^ in ECC, several studies have investigated the implication of mutations in calcium-related genes in HCM pathogenesis. The *TNNC1* gene was among the first genes encoding a Ca^2+^-sensitive/handling protein to be neatly linked to HCM [70]. Subsequently, several mutations in other genes, such as *ALPK3*, *RYR2*, *PLN*, and *JPH2*, have been implicated in the pathogenesis of HCM [67]. It should be noted that sarcomere mutations in HCM either directly or indirectly influence intracellular calcium [71]. The HCM-associated genes linked to calcium with their main functions are summarized in Table 2.

#### 3.2.1. Troponin C1

Troponin is a central regulatory protein of striated muscle contraction located on the actin filament. The cardiac troponin complex is a heterotrimeric myofilament composed of three subunits: an elongated troponin T subunit (TNNT2), an inhibitory troponin I subunit (TNNI3), and a Ca^2+^-sensitive troponin C subunit (TNNC1). TNNC1 is a sarcomeric Ca^2+^ sensor that binds to the cytosolic divalent cation at the specific Ca^2+^ binding site to enhance its interaction with TNNI3. This complex reduces the inhibitory function of TNNI3, releasing it from actin, and causes the troponin–tropomyosin complex to move into the actin groove, exposing myosin binding sites [72]. Accordingly, *TNNC1* plays a critical molecular role in the initiation of myofilament contraction [67]. 

Mutations in the *TNNC1* gene are rare, occurring in ~0.4% of HCM patients [73]. The first HCM-associated mutation in *TNNC1*, c.86T>A; p.(Leu29Gln), was identified by Hoffmann et al. in 2001 [70]. Using in vitro and in situ models, this mutation was found to affect the Ca^2+^-dependent structural change in cardiac TnC in trabeculae under basal conditions and abolish the effect of force-generating myosin cross-bridges. [74]. Six additional mutations in the *TNNC1* gene (c.23C>T; p.(Ala8Val), c.91G>T; p.(Ala31Ser), c.251G>A; p.(Cys84Tyr), c.402G>T; p.(Glu134Asp), c.435C>A; p.(Asp145Glu), and c.363dupG; p.(Gln122Alafs*30)) have been associated with HCM by the screening of the *TNNC1* gene in a cohort of 1025 HCM patients [73,75,76,77]. 

Functional studies showed an increased Ca^2+^ sensitivity of force development for c.23C>T, c.251G>A, and c.435C>A and force recovery for c.23C>T and c.435C>A mutations. The *TNNC1* c.402G>T mutation showed no changes in these parameters [73]. The frameshift mutation *TNNC1* c.363dupG is located in the EF-hand 3 domain and was found to destroy the H-helices of troponin C that are required for the interaction with troponin I [78]. Moreover, the functional analysis suggested that the *TNNC1* c.91G>T mutation directly affects Ca^2+^ sensitivity and may alter Ca^2+^ handling, leading to arrhythmogenesis [67].

#### 3.2.2. Ryanodine Receptor Type 2

Mutations in the ryanodine receptor type 2 (*RYR2*) gene are typically associated with CPVT, ventricular arrhythmias due to calcium release deficiency syndrome, and arrhythmogenic right ventricular cardiomyopathy/ dysplasia (Table 1). In 2006, Fujino et al. reported the first *RYR2* mutation potentially involved in HCM: c.3320C>T; p.(Thr1107Met) [79]. This mutation was subsequently identified in exome-sequencing cohorts with a relatively high frequency (MAF=0.0004) [80] and in CPVT patients [81], questioning its involvement in HCM. Recently, Alvarado et al. (2019) identified a novel *RYR2* mutation c.3372G>A; p.(Pro1124Leu), in an HCM patient who did not have a sarcomeric mutation [82]. Functional studies have shown that homozygous mice for this mutation presented mild cardiac hypertrophy combined with an increase in the expression of calmodulin, a classical inhibitor of RYR2 [82]. 

#### 3.2.3. Alpha Kinase 3

The alpha kinase 3 (*ALPK3*) gene encodes the alpha-protein kinase 3 that may act as a transcriptional regulator through the phosphorylation of cardiac transcription factors [83]. The *ALPK3* gene is early expressed in the cardiac crescent and later remains highly expressed in cardiomyocytes throughout life [84,85]. Recently, *ALPK3* was identified as an important cardiac pseudokinase that inserts into the nuclear envelope and M-band of the sarcomere [85].

Homozygous *ALPK3*-truncating mutations were initially associated with early-onset cardiomyopathy [84]. Pediatric cases reported thereafter consistently displayed a severe clinical presentation with irregular cardiac remodeling and an inconsistent syndromic pattern [84,86,87,88]. Indeed, pediatric cases with biallelic *ALPK3*-truncating mutations showed a variable HCM phenotype with atypical distribution of hypertrophy (concentric/ apical/ asymmetric septal hypertrophy/right ventricular dysfunction) and variable facio-thoraco-skeletal features [84,85,86,87,88,89]. It is noteworthy that homozygous *ALPK3* carriers diagnosed in utero or at birth presented a mixed DCM/HCM phenotype with progression to an HCM phenotype with age [87,89]. Although the majority of heterozygous parents or relatives of the reported cases were found to be healthy or with mild HCM, recent case reports and studies extended the phenotype and genotype spectrum of *ALPK3* mutations to include (i) missense mutations, (ii) compound-heterozygous and autosomal-dominant patterns of inheritance, and (iii) adult-onset HCM with a less severe clinical presentation [89,90].

The *ALPK3* gene appears to be essential for the normal formation of the intercalated disc and the organization of cardiomyofibril in humans and mice [86,88,91]. It has been shown that hiPSCs-derived cardiomyocytes containing a homozygous *ALPK3*- truncating mutation displayed defective Ca^2+^ handling in addition to sarcomeric disorganization and impaired intercalated disc integrity [84,88].

A functional study of the Ca^2+^ flux during contraction demonstrated that *ALPK3*-mutant cardiomyocytes displayed several changes in intracellular Ca^2+^ including significantly increased irregular Ca^2+^-transients, which may explain the cellular hypertrophy [88]. Indeed, using multi-electrode array analysis, *ALPK3*–hiPSCs-derived cardiomyocytes demonstrated an extended extracellular field potential duration, indicating that the loss of *ALPK3* disrupts membrane repolarization [88]. 

These results suggest that intracellular Ca^2+^ is elevated in *ALPK3*-deficient cardiomyocytes, agreeing with what has been observed in other hiPSC models of HCM [84,88]. 

#### 3.2.4. Junctophilin 2

The junctophilin 2 (*JPH2*) gene encodes junctophilin 2, a major component of the junctional membrane complex involved in Ca^2+^ homeostasis and ECC [92]. Junctophilins are a family of proteins found in all excitable cells [93]. *JPH2* plays a crucial role in maintaining the proper structure of the cardiac dyad, which is necessary for effective CICR [94]. The implication of the *JPH2* gene in HCM was first reported by Landstrom et al. (2007) with the identification of three mutations (c.301A>C; p.(Ser101Arg), c.421T>C; p.(Tyr141His), and c.494C>T; p.(Ser165Phe), in three unrelated patients with HCM negative for sarcomeric or Z-disc mutations [95]. The characterization of these mutations using an in vitro model of myocyte culture showed a decrease in CICR amplitude and disruption of cellular ultrastructure. The c.421T>C and c.494C>T mutations were found to induce cellular hypertrophy [95]. Of note, mutations in *JPH2* are considered a rare cause of HCM and are found in less than 1% of index cases [67]. 

#### 3.2.5. Phospholamban

The phospholamban (*PLN*) gene encodes phospholamban, a major substrate for the cAMP-dependent protein kinase in cardiac muscle. In its unphosphorylated state, PLN is an inhibitor of cardiac muscle SERCA2a. This inhibition is abolished upon phosphorylation of the PLN protein. The consecutive activation of the Ca^2+^ pump results in enhanced muscle relaxation rates. Thus, PLN is a key regulator of cardiac diastolic function [96,97,98]. 

Mutations in the *PLN* gene are known to cause DCM [99]. However, rare promoter mutations have been identified in multiple independent cohorts of HCM patients [100,101]. Nevertheless, only the rare truncating *PLN* c.116T>G; p.(Leu39Ter) mutation is recognized as a causative HCM mutation [102]. This mutation cosegregated with HCM in a multigenerational family and the truncated protein is likely to impair PLN and SERCA2a interactions [103]. The authors also estimated an overall yield of *PLN*–HCM mutations of 0.65% by comparing different studies reporting *PLN* mutations in HCM cohorts [102].

**Table 2:** Calcium-related genes linked to hypertrophic cardiomyopathy.

HGNC_Gene Symbol	Full Name	Location	Exon Count	Protein Function	OMIM Disease	OMIM IDs	Inheritance	References
*RYR2*	Ryanodine receptor 2	1q43	105	Regulates muscle hypertrophy and actin filament-based movement.	ARVD2	600996	AD	[104,105]
					VA	115000	AD	
					CPVT1	604772	AD	
*ALPK3*	Alpha kinase 3	15q25.3	14	Striated muscle cell development and differentiation. Actomyosin structure organization. Heart development and morphogenesis. Regulates the expression and localization of critical proteins in both the sarcomere M-band and nuclear envelope of cardiomyocytes.	HCM,27	618052	AR	[84,85]
*TNNC1*	Troponin C1, slow skeletal and cardiac type	3p21.1	6	Ca^2+^ sensor and key regulator of cardiac contraction. Its Ca^2+^-binding properties modulate the rate of cardiac muscle contraction at submaximal levels of Ca^2+^ activation. Modulates the Ca^2+^-binding properties. Calcium-binding subunit of the troponin complex responsible for initiating striated muscle contraction in response to calcium influx.	DCM, 1Z	611879	AD	[106,107,108]
					HCM13	613243	AD	
*JPH2*	Junctophilin 2	20q13.12	5	Mediates cross-talk between the cell surface and ER. Cellular Ca^2+^ signaling in excitable cells form junctional membrane complexes between the plasma membrane and the ER/SR.	DCM, 2E	619492	AR	[109,110,111]
					HCM,17	613873	AD	
*PLN*	Phospholamban	6q22.31	2	Crucial Ca^2+^ cycling protein and a primary mediator of the beta-adrenergic effects, resulting in enhanced cardiac activity. Regulates the activity of the sarcoplasmic Ca^2+^ ATPase isoform, a regulator of the kinetics of cardiac contraction. Unphosphorylated PLN reduces ATP2A1 affinity for Ca^2+^ and affects enzymatic turnover.	DCM, 1P	609909	AD	[97,112,113]
					HCM, 18	613874	AD	

ARVD: arrhythmogenic right ventricular dysplasia; AD: autosomal dominant; AR: autosomal recessive; CPVT: catecholaminergic polymorphic ventricular tachycardia; DCM: dilated cardiomyopathy; ER: endoplasmic reticulum; SR: sarcoplasmic reticulum; HCM: hypertrophic cardiomyopathy; LQTS: long QT syndrome; VA: ventricular arrhythmias due to cardiac ryanodine receptor calcium release deficiency syndrome.

## 4. Conclusions

This review aimed to summarize the Ca^2+^-related genes linked to CPVT and HCM with their underlying physiopathological mechanisms. Reporting HCM patients with mutations in Ca^2+^-related genes stems from the fact that abnormal Ca^2+^ handling is a common feature of structural heart diseases. Studying calcium handling in healthy and diseased hearts may improve our understanding of Ca^2+^-mediated cardiac diseases and aid the advancement of therapeutic strategies.

## Data Availability

Not applicable.
